# Malignant fibrous histiocytoma of the heart: a rare case report of an aggressive cardiac sarcoma mimicking myxoma

**DOI:** 10.1093/ehjcr/ytaf080

**Published:** 2025-02-14

**Authors:** Youssef Ahmad, Tumodir Abdallah, Modar AlAli, Monaza Adeeb, Majdi Alhammoud

**Affiliations:** Faculty of Medicine, Tartous University, Tartous, Syrian Arab Republic; Faculty of medicine, Al Quds university, University Street, abu Dies, Jerusalem 144, Palestine; Faculty of Medicine, Damascus University, Damascus 30621, Syrian Arab Republic; Tbilisi State Medical University, Vazha-Pshavela Ave, 33, Tbilisi 0186, Georgia; Al-Assad University hospital, Damascus 30621, Syrian Arab Republic

**Keywords:** Primary cardiac tumour, Malignant fibrous histiocytoma, Cardiac sarcoma, Myxoma-mimicking malignancy, Diagnostic challenges, Case report

## Abstract

**Background:**

Primary cardiac tumours are rare, with an incidence ranging from 0.001% to 0.03%. Among these, sarcomas constitute the majority of malignant cases, with malignant fibrous histiocytoma (MFH) being one of the least commonly reported and most aggressive forms.

**Case summary:**

We present the case of a 40-year-old female who experienced non-specific symptoms, including malaise, dyspnoea, and recurrent chest pain. Initial investigations, including echocardiography, revealed a large mass in the left atrium, initially diagnosed as a myxoma. However, histopathological examination following surgical resection confirmed the diagnosis of MFH. Despite surgical intervention and chemotherapy, the tumour recurred, resulting in the patient's death 2 months after surgery.

**Discussion:**

Cardiac sarcomas, particularly MFH, pose significant diagnostic challenges due to their rarity and non-specific symptoms. These malignant tumours often mimic benign conditions, clinically and diagnostically, in modalities such as transoesophageal echocardiography and transthoracic echocardiography. While advanced imaging techniques are critical for accurate diagnosis, socioeconomic factors may limit access. Histopathological examination remains the gold standard for definitive diagnosis. The aggressive nature of these tumours, characterized by rapid recurrence and poor prognosis, highlights the need for improved diagnostic and therapeutic strategies. Future research should focus on targeted therapies and immunotherapy, which promise to improve outcomes in managing these malignancies.

**Learning points**

- Malignant fibrous histiocytoma (MFH) is a rare and aggressive primary cardiac tumour that poses significant diagnostic challenges due to its non-specific symptoms and ability to mimic benign conditions, underscoring the need for comprehensive diagnostic approaches, including histopathological examination.
- Despite complete surgical resection, MFH has a high recurrence rate and poor prognosis, highlighting the importance of combining surgery with adjuvant therapies and exploring novel treatments like targeted therapies and immunotherapy for better patient outcomes.

## Introduction

Primary cardiac tumours are exceptionally rare, with most being benign.^1^ Among malignant cases, sarcomas, including the rare malignant fibrous histiocytoma (MFH), represent an exceedingly rare and aggressive subset.^2,3^ Sarcomas are difficult to diagnose in early stages due to their rare incidence and late-presenting symptoms, often leading to a poor prognosis. Biopsy of the mass with specific histopathological staining remains the favourable diagnostic method since it allows for a definitive diagnosis.^4^ Treatment remains challenging, with complete surgical resection being the gold standard. However, appropriate chemotherapy should be administered following the surgical procedure.^5,6^ We present a case of a 40-year-old female initially suspected of having a benign cardiac myxoma but later diagnosed with a rare MFH of the left atrium (LA).

## Summary figure

**Table:** 

Day/period	Events
Day 0	Patient presents with malaise, dyspnoea, and recurrent chest pain. Physical exam: Systolic heart murmur detected, NYHA class II dyspnoea. Initial diagnostics: ECG: Normal sinus rhythm, left axis deviation, poor R wave progression, rS pattern in lead III and aVF. Chest X-Ray: Mild bilateral hilar congestion, normal cardiac index.
Day 1–3	Echocardiography: Discovery of a large (3.43 × 3.47 cm) heterogeneous, lobulated mass in the left atrium. Central mitral valve regurgitation noted.
Day 4–7	Surgical intervention: Emergent surgery performed; intraoperative findings include diffuse atrial wall thickening. Frozen sections suggest malignancy. Histopathological examination: Diagnosis of malignant fibrous histiocytoma confirmed.
Post-surgery—Day 7	Post-operative computed tomography scan and X-ray: No significant findings except for bilateral pleural effusion and atelectasis in the right lung. Oncology referral: Patient starts chemotherapy.
Follow-up—2 months later	Tumour recurrence observed; unfortunately, the patient succumbs to the disease.

## Case presentation

**Figure 1:**
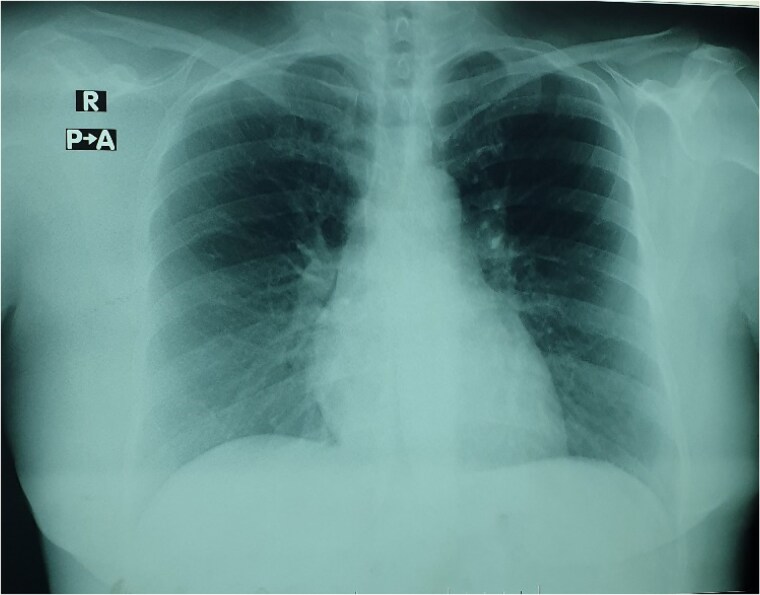
An X-ray before surgery showing bilateral hilar congestion.

**Figure 2:**
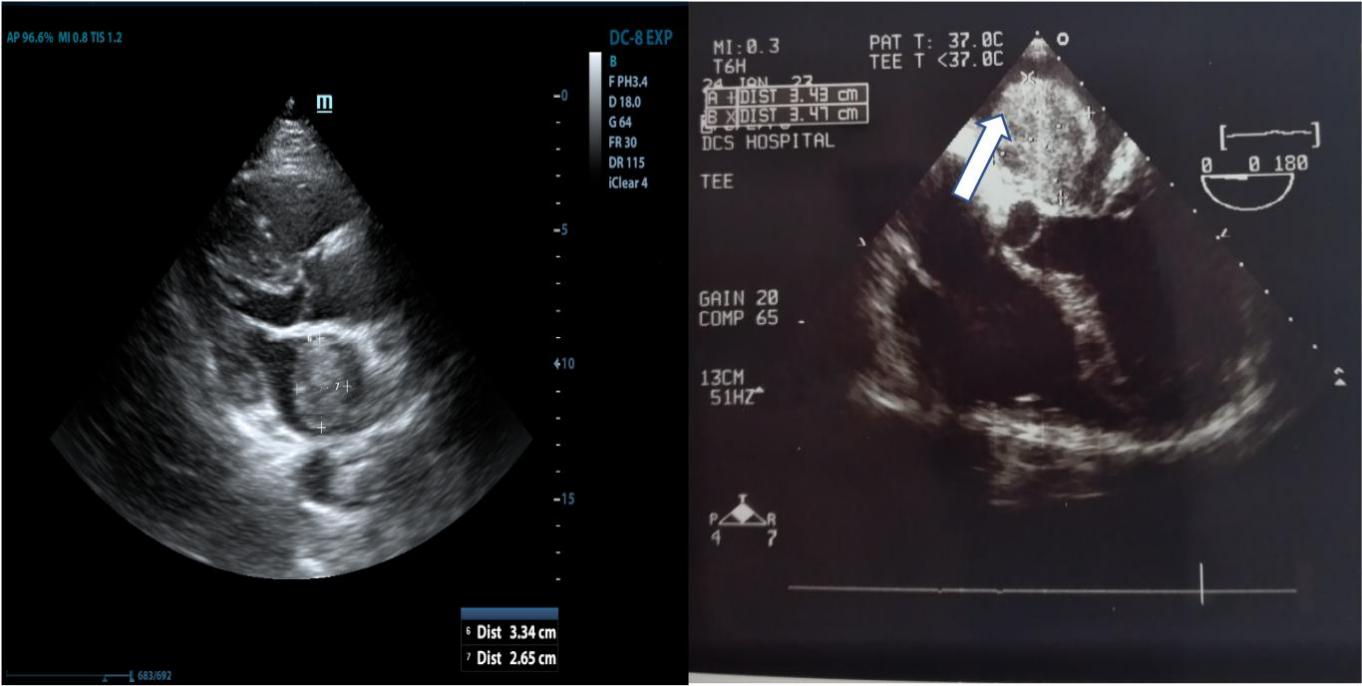
Left photo: Transoesophageal echocardiography showing the mass in left atria not prolapsing through the mitral valve. Right photo: Transoesophageal echocardiography showing a heterogeneous mass in left atria with a broad-based attachment to the atrial septum (arrow).

**Figure 3:**
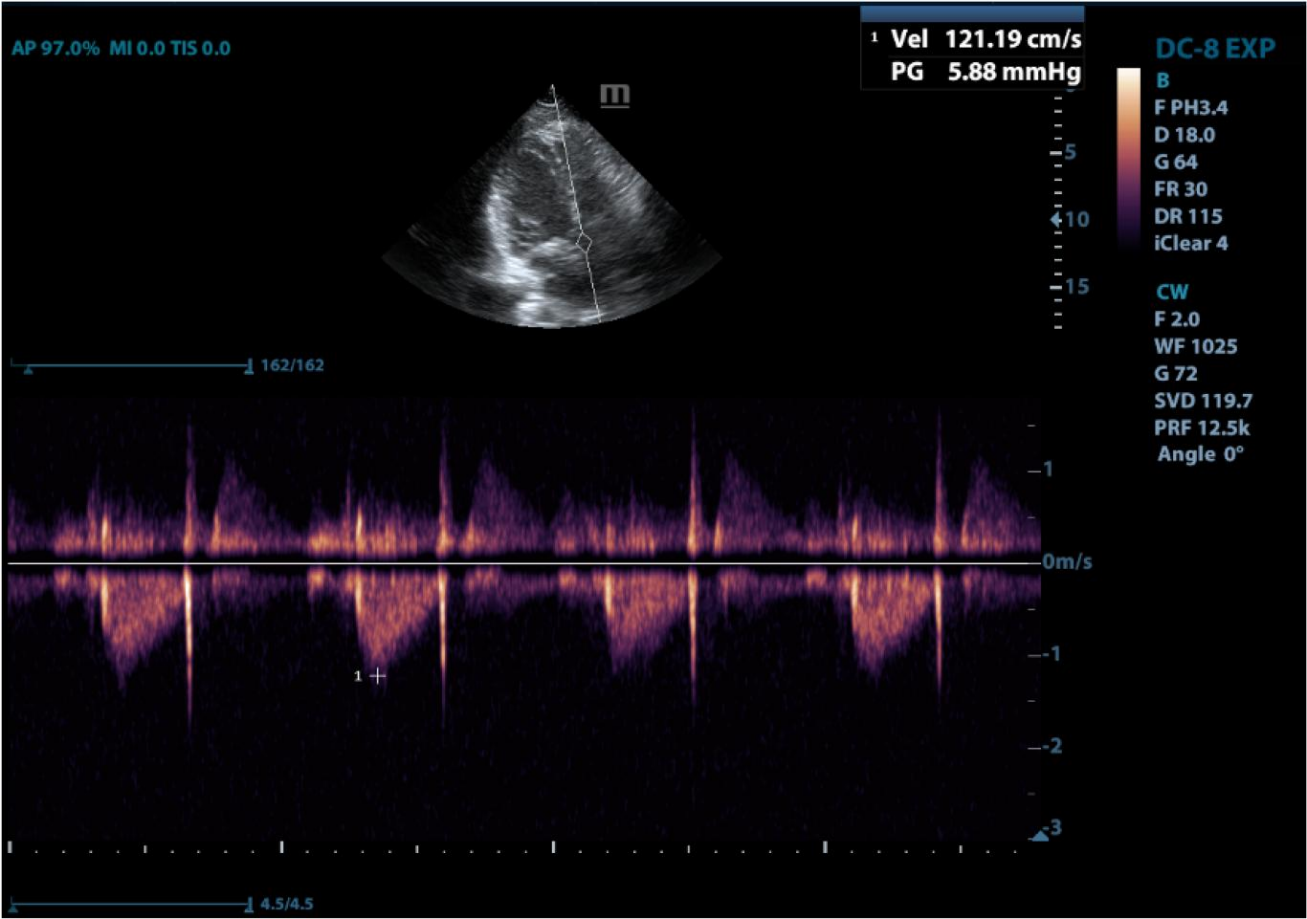
A transoesophageal echocardiography showing the regurgitation in the mitral valve.

A 40-year-old female presented to the hospital with malaise, dyspnoea, multiple episodes of syncope, and recurrent chest pain. Physical examination revealed a systolic heart murmur at the fifth left intercostal space at the midclavicular line and mild dyspnoea (New York Heart Association Class II). The patient had type 2 diabetes mellitus with no other significant medical or family history. ECG showed normal sinus rhythm. The initial chest X-ray found mild bilateral hilar congestion with a normal cardiac index (*Figure 1*). Transthoracic and subsequent transoesophageal echocardiography showed a heterogeneous, lobulated, pedunculated large mass in the LA measuring 3.43 × 3.47 cm with a broad-based attachment to the atrial septum (*Figure 2A* and *B*). The mass surface was moving towards the mitral valve during diastole without prolapsing through the mitral valve or obstructing diastolic flow. The ultrasound study also showed central mitral valve regurgitation (1.5/4) with a dilated LA (44 mm in systole) (*Figure 3*). It also reported normal ventricular volumes (end diastolic volume [EDV] = 126.59 mL, ejection fraction [EF] = 67%), systolic pulmonary pressure of 35 mmHg, and mild tricuspid regurgitation (<1/4). There were no intracardiac thrombi or pericardial effusions observed.

The diagnosis was presumed to be a myxoma, and an emergent surgical removal was planned to prevent secondary complications. During the surgery, after opening the LA, diffuse thickening in the atrial walls was found, raising suspicion of malignancy. Examination of the intraoperative frozen sections from the mass supported these suspicions as well. The mass was removed and sent for histopathologic examination.

**Figure 4:**
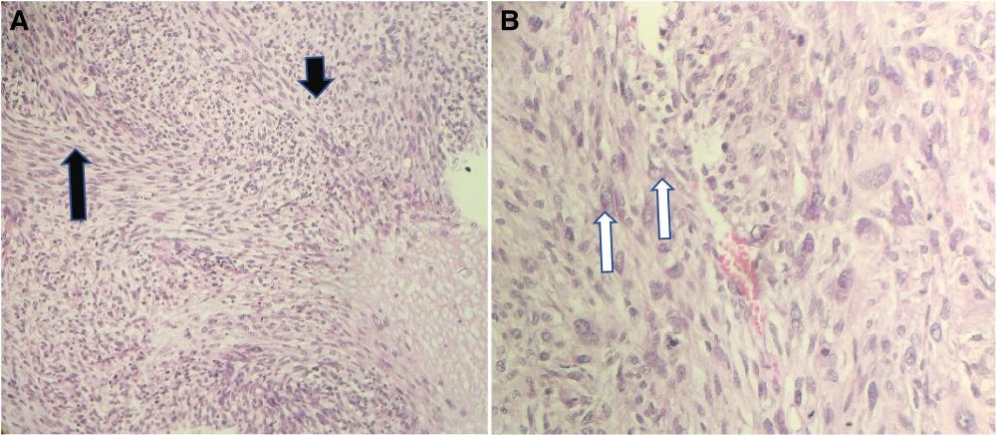
A high power magnification showing storiform patterns (black arrows). (*B*) High power magnification showing nuclear pleomorphism with bizarre nuclei (white arrows).

Haematoxylin-eosin staining showed a high-grade spindle cell sarcoma, favouring (undifferentiated high-grade pleomorphic sarcoma) MFH, G3, showing storiform patterns with irregular fascicles, hypercellularity, bizarre nuclei, and some giant cells (*Figure 4A* and *B*). Further immunohistochemistry (IHC) studies were performed confirming the previous diagnosis. A computed tomography scan and chest X-ray were performed 3 days after surgery, exhibiting no mediastinal or axial lymph node enlargement. The patient was prescribed bisoprolol fumarate and apixaban, in addition to continuing metformin, which had been taken previously. She was referred to the oncology department to begin chemotherapy. A follow-up check revealed the recurrence of the tumour with a pedunculated LA septal mass with a significant weight loss after the surgery. Unfortunately, the patient died 2 months later.

## Discussion

Primary cardiac sarcomas (PCSs) are rare and aggressive tumours with a poor prognosis, exhibiting a median survival of 9 to 27 months, affecting both sexes equally. The most frequent types are angiosarcomas (37%), undifferentiated sarcomas (24%), leiomyosarcoma (8%–9%), and osteosarcoma (3%–9%).^5,7,8^ High-grade spindle cell sarcoma primarily affects individuals aged 20–50 years. Specifically, MFH represents a smaller subset (11%–24%) within this existing rare category.^7^ Aggressive cardiac sarcomas (highly malignant heart tumours) include subtypes like MFH, now classified as undifferentiated pleomorphic sarcoma (UPS). Both MFH/UPS and high-grade spindle cell sarcoma describe tumours with spindle-shaped and pleomorphic cells, reflecting their aggressive nature and histological overlap.

Clinical presentation can range from an asymptomatic incidental finding to one of the well-known triad of obstructive, embolic, and systemic symptoms (pyrexia, anorexia, arthralgia, and weight loss).^3,7,9^ In rare instances, sarcomas may also present as a pseudoaneurysm, with one such case reported.^2^ In our case, the patient exhibited non-specific symptoms such as malaise, dyspnoea, and chest pain, commonly seen in more benign conditions like atrial myxomas. The differential diagnosis is broad, including primary benign or malignant cardiac tumours, clots, vegetations, metastatic tumours, or collagen vascular diseases.^7,9^

For diagnosis, echocardiography is the first-line imaging technique with good sensitivity and specificity (95%–100%). Transthoracic echocardiography (TTE) provides information about the location, morphology, mobility, and haemodynamic impact. Transoesophageal echocardiography (TEE) is superior to TTE and demonstrates how the surrounding tissue and the mass relate to one another. They often establish the initial diagnosis and direct further investigation. Computed tomography offers insights into tumour staging, calcification, adipose content, and vascularity. Positron emission tomography (PET), (18F-FDG-PET) distinguishes benign from malignant cardiac tumours by assessing metabolic activity and provides very accurate staging and prognosis of disseminated disease.^3,9^ However, in our case, PET could not be utilized due to socioeconomic constraints. This limitation emphasizes the importance of accessible and comprehensive diagnostic facilities for accurate diagnosis.

Histopathological examination remains crucial for definitive diagnosis and classification. IHC offers guidance on prognosis, treatment, and recurrence, which emphasize the expression of regulatory proteins and cell kinetics.^3,5^ In this case, initial TTE and TEE findings suggested a benign myxoma, but biopsy and histopathology revealed a high-grade spindle cell sarcoma, emphasizing the importance of considering malignancy even with benign-appearing imaging results.

Complete surgical excision significantly improves survival in cardiac tumour patients. A cohort study of 162 cardiac tumour cases reported cumulative survival rates of 94% at 30 days, 85% at 1 year, 72% at 5 years, and 59% at 15 years post-surgery.^10^ Another study on 335 patients with PCSs found 1-year all-cause mortality and cancer-specific mortality rates of 49.0% and 42.1%, respectively, with better outcomes in those undergoing surgery combined with chemotherapy.^11^

Adjuvant chemotherapy or radiation offers additional survival benefits. Retrospective analyses suggest combinations of anthracyclines and ifosfamide improve outcomes for high-grade sarcomas like UPS. A meta-analysis of 14 perioperative chemotherapy studies found modest survival benefits for patients receiving adjuvant therapy. Adding doxorubicin and ifosfamide reduced the absolute risk of death by 11% compared with those without adjuvant therapy.^12,13^ Low-dose radiation therapy, administered pre- or post-surgery, further enhances outcomes when combined with surgical resection.^3,6^

Future management of rare aggressive tumours may focus on targeted therapies and improved diagnostics for early detection. Crombe *et al*.^14^ discuss tumour antigens in sarcomas that immunotherapy can target and scattered T cells in tumours, supporting immunotherapy as a promising treatment option. In conclusion, this case illustrates the aggressive nature of MFH, marked by rapid recurrence (within 2 months post-surgery), poor prognosis, and limited response to conventional treatments, while highlighting the diagnostic complexities of this rare cardiac tumour and underscoring the need for comprehensive diagnostic approaches and ongoing research into more effective therapeutic strategies.

## Data Availability

All data generated or analysed during this study are included in this published article (and its supplementary information files).

## Lead author biography

Dr Youssef Ahmad is a medical graduate from the University of Tartous, Syria, where he earned his medical degree (MD). He has conducted research in various areas of cardiology, with several publications in fields such as cardiothoracic surgery, congenital heart disease, and coronary artery disease.

## Notes

**Consent:** Written informed consent was obtained from the patient and their legal guardian for the publication of this case report and any accompanying images. This study complies with the ethical guidelines outlined by the Committee on Publication Ethics (COPE).

**Funding:** None declared.
